# The prognosis of MG patients with different thymic pathology: a multicenter retrospective cohort study

**DOI:** 10.1186/s12916-025-04509-w

**Published:** 2025-12-16

**Authors:** Moli Fan, Hao Zhang, Yutong Shi, Xiaoyu Huang, Ying Cui, Zihao Yu, Xiao-He Zhang, Xiao-Jing Zhang, Ying-Ping Xue, Lei Huang, Fu-Dong Shi, Guo-Yan Qi

**Affiliations:** 1 Department of Neurology, Tianjin Medical University General Hospital, Tianjin, China; 2 China National Clinical Research Center for Neurological Diseases, Beijing Tiantan Hospital, Capital Medical University, Beijing, China; 3 First Hospital Shijiazhuang, Treatment Center of Myasthenia Gravis, Shijiazhuang, Hebei People’s Republic of China

**Keywords:** Myasthenia gravis, Thymectomy, Thymus pathology

## Abstract

**Background:**

To identify the associations between thymic pathology and the prognosis of myasthenia gravis (MG) patients.

**Methods:**

In this multicenter retrospective study, 1,254 myasthenia gravis (MG) patients who underwent thymectomy across four clinical centers were included. Participants were categorized by thymic pathology into thymomatous and non-thymomatous groups. Primary outcome was postoperative deterioration. Secondary outcomes comprised the proportion of patients achieving minimal manifestation status (MMS) within the first year after surgery and conversion from ocular MG (OMG) to generalized MG (GMG) within 2 years of symptom onset. Subgroup analyses assessed associations between world health organization (WHO) pathological type (both groups) or Masaoka stage (thymoma patients) and prognosis.

**Results:**

Thymomas were associated with an increased risk of deterioration in both Cox regression (adjusted HR = 1.40 [1.18, 1.66], *p* < 0.001) and logistic regression analyses (1-year deterioration: adjusted OR = 1.59 [1.15, 2.20], *p* = 0.005; 3-year deterioration: adjusted OR = 1.40 [1.01, 1.94], *p* = 0.047). Additionally, thymomas were linked to a higher conversion rate (adjusted OR = 2.37 [1.15, 4.86], *p* = 0.019). However, thymoma showed no significant association with MMS (adjusted *p* = 0.682). In the thymoma subgroup, neither pathological type nor Masaoka stage was significantly associated with deterioration (pathological type: 1-year *p* = 0.069, 3-year *p* = 0.220; Masaoka stage: 1-year *p* = 0.944, 3-year *p* = 0.909), first-year MMS attainment (pathological type: *p* = 0.067; Masaoka stage: *p* = 0.579), or conversion rate (pathological type: *p* = 0.606; Masaoka stage: *p* = 0.163). Similarly, in the nonthymomatous group, WHO pathological type was not significantly correlated with deterioration (1-year *p* = 0.806, 3-year *p* = 0.654), MMS achieved (*p* = 0.940), or conversion (*p* = 0.755).

**Conclusions:**

This study demonstrated an association between thymoma and higher risks of clinical deterioration, which was independent of WHO pathological type or Masaoka stage.

## Background

Thymectomy is an important therapeutic approach for MG patients, both with and without thymoma [1, 2]. However, the relationship between thymic pathology and prognosis remains controversial. Many studies have suggested that thymomas lead to worse outcomes, including poorer recovery after thymectomy [3, 4] and a higher conversion rate from OMG to GMG [5–8]. Interestingly, other conclusions have been drawn in some reports. For example, many studies have indicated that there is no significant difference in prognosis between thymomatous and nonthymomatous pathologies [9–12]. Additionally, several studies have suggested that thymic hyperplasia is associated with a higher relapse rate [13] or that thymoma influences prognosis only in the short term rather than in the long term [14]. Most of the above studies are limited by small sample sizes, single-center studies or studies that focus on only one outcome. Thus, more comprehensive studies are needed.

Furthermore, specific WHO pathological types and Masaoka stages, which describe the relationships between thymomas and surrounding organs, are used to classify the severity of thymomas. Otherwise, the pathologist records the nonthymomatous pathology as thymic hyperplasia, a thymic cyst, thymic atrophy or a normal thymus. The impact of these WHO pathologies, or the Masaoka stage, on prognosis remains a topic of debate. For thymoma pathology, scientists who support different specific pathological types [15–18] or Masaoka stages [15, 19–21] have different outcomes, and those who hold diverse opinions that specific pathological types [14, 22, 23] or Masaoka stages [14, 18, 22] do not contribute to prognosis are both large in number. For nonthymomatous pathologies, conflicting findings also exist [13, 24], and there is not enough evidence to demonstrate the prognosis of patients with different nonthymomatous WHO pathological types [5].

In summary, the prognoses of different thymic pathologies are still unclear. Large, multicenter studies that consider various outcomes, including comparisons between thymomatous and nonthymomatous MG patients and prognoses according to different WHO pathological types or Masaoka stages, are needed to provide evidence for clinical practice.

## Methods

### Study design and participants

**Fig. 1:**
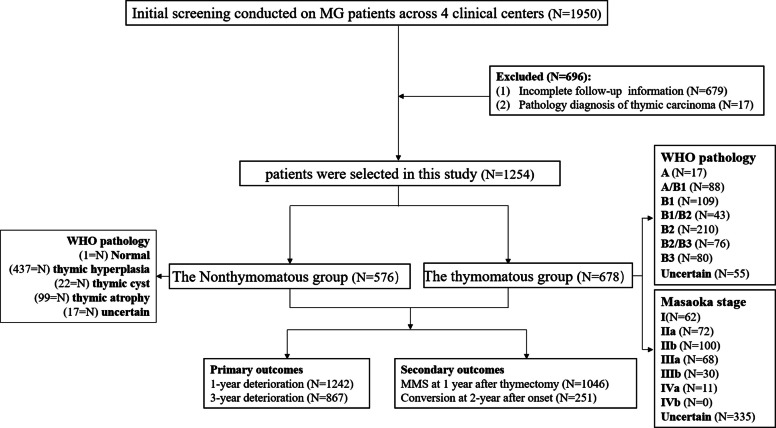
The flowchart to illustrate the patient selection process

This retrospective study included 1,254 patients from 4 centers, Tianjin Medical University General Hospital, Beijing Tiantan Hospital, Shijiazhuang People’s Hospital and Changzhou First People’s Hospital, between January 2020 and December 2023. The inclusion criteria were as follows: (1) clinically diagnosed with MG after 2000; (2) underwent thymectomy after MG diagnosis; and (3) had accessible operation, pathology and follow-up data. Exclusion criteria were (1) incomplete information or (2) a diagnosis of thymic carcinoma. (Fig. 1).

Our study specifically excluded thymic carcinoma (Type C thymoma). This decision was based on two primary reasons. First, thymic carcinoma is clinically distinct from thymoma, characterized by a higher recurrence rate and a relative lower 5-year survival rate. Second, the treatment paradigm for thymic carcinoma is primarily focused on oncological management rather than myasthenia gravis (MG) control. This introduces numerous potential confounding factors that are difficult to adjust for. Therefore, to ensure a more homogeneous cohort, we excluded these patients.

The collected patient data included demographics, primary symptoms, antibody type, worst Myasthenia Gravis Foundation of America clinical classification (MGFA) type before surgery, perioperative immunoregulatory medication (IM) history, primary onset-thymectomy time, thymic pathology, perioperative MG crisis, postoperative immunoregulation drug history, deterioration condition during different periods, deterioration conditions at different time points, MMS achieved in the first year after thymectomy, and conversion condition within 2 years after symptom onset.

### Outcomes

The primary outcome was deterioration, defined as (1) new MG symptoms and (2) worse MGFA classification. Secondary outcomes include MMS in the first year after thymectomy and the conversion rate from OMG to GMG within 2 years after symptom onset. Particularly for the conversion outcome, the variable “onset-thymectomy time” was recoded based on the temporal sequence between thymectomy and conversion. Patients who underwent thymectomy before conversion occurred were labeled “Yes”, while those who did not were labeled “No”.

### Subgroups

In our study, we divided the patients into two subgroups on the basis of pathology: (1) the thymoma subgroup and (2) the nonthymomatous subgroup. The associations between specific WHO pathological types or Masaoka stages of thymoma and prognosis were further explored within each subgroup.

### Statistical analysis

Categorical variables were compared between groups via the chi-square test or Fisher’s exact test. Quantitative data are expressed as medians (Min, Max) and were analyzed via the Mann‒Whitney test. Variables with a *p*-value < 0.1 from the univariate analysis and known to be associated with prognosis based on existing literature were included as potential confounders in the multivariate model. Simple and multivariate Cox/logistic regression models were used to identify differences between the groups for all outcomes. A linear/box diagram was used to visualize comparative trends. Cases with incomplete data for the variables relevant to a specific outcome were excluded from that particular analysis (case-wise deletion). The same statistical analysis was also used for all subgroups, and all the statistical analyses were performed with SPSS version 24.0. *p* < 0.05 was assumed to indicate significant results for all tests.

## Results

### Demographics and clinical characteristics

A total of 1,254 patients were selected for this study according to our inclusion and exclusion criteria. A total of 678 patients (54.1%) were included in the thymomatous group, and 576 were included in the nonthymomatous group. Eighteen patients had negative antibody results after more than 2 tests, and 17 patients had an unknown antibody type because of missing data. Six patients in the nonthymomatous group were Musk antibody positive, and 1 patient in the thymomatous group was positive only for the Titin antibody.

Patient characteristics are summarized in Table 1. Compared with the nonthymomatous group, the thymoma group had a higher age of onset (thymomatous vs. nonthymomatous, 46 vs. 37, *p* < 0.001), a greater percentage of male patients (thymomatous vs. nonthymomatous, 53.2% vs. 43.9%, *p* < 0.001), a lower percentage of other immune disease (thymomatous vs. nonthymomatous, 3.5% vs. 10.2%, *p* < 0.001), and a lower likelihood of receiving immunoregulation medicine both before and after thymectomy (thymomatous vs. nonthymomatous, pre-thymectomy: 23.6% vs.47.9%, *p* < 0.001; post-thymectomy: 44.5% vs.81.1%, *p* < 0.001). Additionally, shorter onset-operation times (thymomatous vs. nonthymomatous, 2 vs. 12, *p* < 0.001) and higher rates of perioperative MG crisis (thymomatous vs. nonthymomatous, 6.6% vs. 2.3%, *p* < 0.001) were observed in thymoma patients. Moreover, 76% of patients in the thymomatous group and only 57.4% of patients in the nonthymomatous group were classified as MGFA class 2b or lower, indicating better MGFA classification in the thymomatous group (*p* < 0.001). Finally, there was no significant difference between the two groups in terms of primary symptoms (*p* = 0.054) or immunoregulatory medicine history within 2 years after onset (*p* = 0.809).

**Table 1:** Clinical characteristics of the patients

	**Total (***N = 1254)*	**Thymomatous (***N*** = 678)**	**Nonthymomatous (***N = 576)*	**P***value*
Age (year)	43 (1,87)	46 (16,87)	37 (1,85)	< 0.001
Gender (male%)	49.0% (614)	53.2% (361)	43.9% (253)	< 0.001
Other immune disease	6.6% (83)	3.5% (24)	10.2% (59)	< 0.001
Primary symptom				0.054
OMG	54.7% (686)	52.2% (354)	57.6% (332)	
GMG	45.3% (568)	47.8% (324)	42.4% (244)	
Antibody type				
AchR +	96.7% (1212)	98.4% (667)	94.6% (545)	< 0.001
MusK +	0.5% (6)	0	1.0% (6)	
Other type +	0.1% (1)	0.1% (1)	0	
Negative	1.4% (18)	0.1% (1)	3.0% (17)	
Unknown	1.4% (17)	1.3% (9)	1.4% (8)	
Worst MGFA before thymectomy				
1	24.8% (311)	33.2% (225)	14.9% (86)	< 0.001
2a	11.5% (144)	11.2% (76)	11.8% (68)	
2b	31.2% (391)	31.6% (214)	30.7% (177)	
3a	4.4% (55)	2.7% (18)	6.4% (37)	
3b	15.6% (196)	14.2% (96)	17.4% (100)	
4a	3.7% (46)	1.8% (12)	5.9% (34)	
4b	4.9% (61)	1.9% (13)	8.3% (48)	
5	4.0% (50)	3.5% (24)	4.5% (26)	
Immunoregulation medicine before thymectomy	34.8% (436)	23.6% (160)	47.9% (276)	< 0.001
Immunoregulation medicine after thymectomy	61.3% (769)	44.5% (302)	81.1% (467)	< 0.001
Immunoregulation medicine in 2 years after onset	41.5% (521)	30.7% (208)	54.3% (313)	0.809
Post-thymectomy radiotherapy	NA	34.2% (232)	NA	NA
Post-thymectomy chemotherapy	NA	15.6% (106)	NA	NA
Primary onset-thymectomy time (month)	4 (1,288)	2 (1,156)	12 (1,288)	< 0.001
Perioperative MG crisis	4.6% (58)	6.6% (45)	2.3% (13)	< 0.001

### Deterioration: thymomatous vs. nonthymomatous

**Fig. 2:**
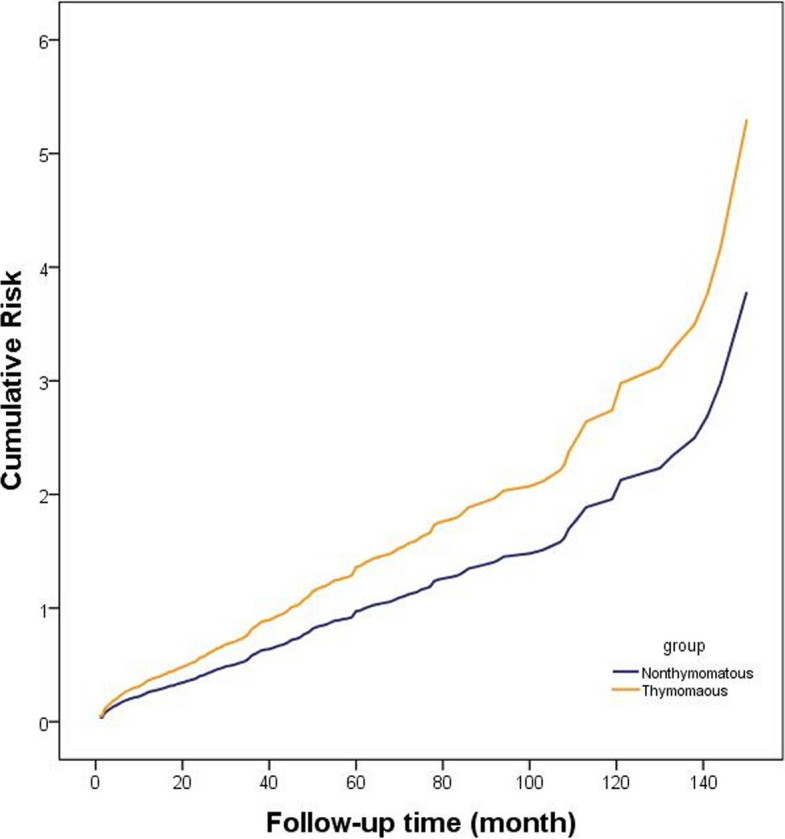
The cumulative risk over time in the different group

For deterioration, in the multivariate Cox regression analysis, the thymoma subgroup exhibited a significantly higher risk of clinical deterioration compared to the non-thymomatous subgroup (HR = 1.40 [1.18, 1.66], adjusted *p* < 0.001), after adjusting for age, sex, coexisting immune diseases, primary symptoms, antibody type, preoperative immunomodulatory therapy, worst preoperative MGFA class, time from onset to thymectomy, perioperative myasthenic crisis, and postoperative immunomodulatory therapy.(Table 2, Fig. 2).

**Table 2:** COX model of the association between thymoma (except thymic carcinoma) and deterioration

	**P**	**OR (95%CI)**	**Adjusted p**	**Adjusted HR (95%CI)**
Age	< 0.001	1.01 (1.00,1.01)	0.797	1.01 (0.99,1.01)
Gender	0.072	0.88 (0.76,1.01)	0.914	0.99 (0.85,1.15)
Other immune disease	0.441	0.89 (0.67,1.19)	0.939	1.01 (0.75,1.36)
Primary symptom	0.103	1.13 (0.98,1.30)	0.899	0.99 (0.85,1.16)
Antibody type	0.375	0.95 (0.84,1.07)	0.672	0.98 (0.87,1.10)
Preoperative IM	< 0.001	0.76 (0.65,0.89)	0.051	1.20 (0.99,1.43)
Worst preoperative MGFA class	< 0.001	0.89 (0.85,0.92)	0.026	0.95 (0.91,0.99)
Primary onset-thymectomy time	< 0.001	0.99 (0.99,0.99)	0.491	0.99 (0.99,1.01)
thymoma	< 0.001	1.79 (1.55,2.08)	< 0.001	1.40 (1.18,1.66)
Perioperative MG crisis	0793	1.04 (0.76,1.43)	0.234	1.22 (0.88,1.68)
Postoperative IM	< 0.001	0.40 (0.35,0.46)	< 0.001	0.44 (0.37,0.52)

**Fig. 3:**
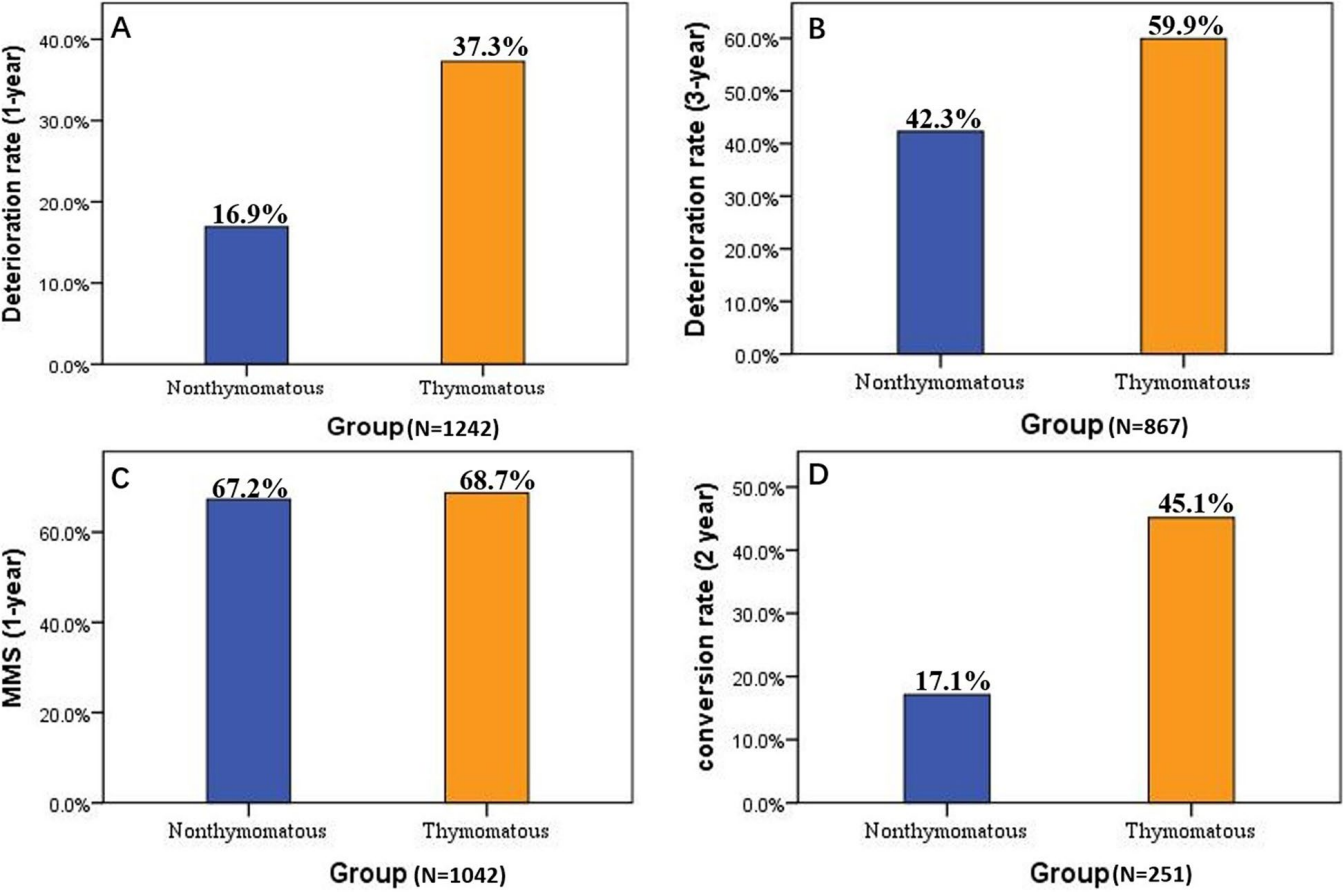
Distribution between the two groups; deterioration condition at 1 year (**A**) and 3 years (**B**) post-thymectomy. MMS in the first year after thymectomy (**C**); conversion rate from OMG to GMG within 2 years after symptom onset (**D**)

In the multivariate Logistic regression analysis, 1,242 patients had follow-up data at 1 year (deterioration rate, Thymomatous group: 37.3%; Nonthymomatous group: 16.9%), and 867 patients had follow-up data at 3 years (deterioration rate, Thymomatous group: 59.9%; Nonthymomatous group: 42.3%) after thymectomy. Compared with nonthymomatous patients, thymomas were associated with a 59% increased risk of deterioration at 1 year after thymectomy and a 40% increased risk at 3 years (1-year deterioration: OR = 1.59 [1.15, 2.20], adjusted *p* = 0.005; 3-year deterioration: OR = 1.40 [1.01, 1.94], adjusted *p* = 0.047). (Table 3, Fig. 3A, B).

**Table 3:** Logistic model of the association between thymoma (except thymic carcinoma) and deterioration in different year

	**P**	**OR (95%CI)**	**Adjusted p**	**Adjusted OR (95%CI)**
**1 year deterioration (***N = 1242)*				
thymoma	< 0.001	2.92 (2.23,3.82)	0.005	1.59 (1.15,2.20)
Gender	0.015	0.74 (0.57,0.94)	0.183	0.83 (0.63,1.09)
Other immune disease	0.297	0.76 (0.45,1.28)	0.842	0.94 (0.53,1.68)
Primary symptom	0.309	0.88 (0.68,1.13)	0.737	1.05 (0.78,1.42)
Antibody type	0.089	0.80 (0.61,1.04)	0.237	0.85 (0.65,1.12)
Preoperative IM	< 0.001	0.50 (0.38,0.67)	0.507	1.12 (0.80,1.57)
Worst preoperative MGFA class	< 0.001	0.83 (0.78,0.89)	0.534	0.97 (0.89,1.06)
Primary onset-thymectomy time	< 0.001	0.99 (0.98,0.99)	0.448	0.99 (0.99,1.01)
age	< 0.001	1.02 (1.01,1.03)	0.262	1.01 (0.99,1.02)
Perioperative MG crisis	0.215	0.66 (0.35,1.27)	0.549	0.81 (0.40,1.63)
Postoperative IM	< 0.001	0.19 (0.14,0.24)	< 0.001	0.23 (0.17,0.32)
**3-year deterioration (***N = 867)*				
thymoma	< 0.001	2.04 (1.55,2.68)	0.047	1.40 (1.01,1.94)
Gender	0.408	0.89 (0.68,1.17)	0.818	1.04 (0.77,1.39)
Other immune disease	0.457	0.82 (0.48,1.40)	0.666	0.88 (0.49,1.57)
Primary symptom	0.358	0.88 (0.67,1.15)	0.864	1.03 (0.75,1.41)
Antibody type	0.273	0.89 (0.74,1.10)	0.666	0.96 (0.78,1.78)
Preoperative IM	0.179	0.82 (0.61,1.10)	0.014	1.54 (1.09,2.17)
Worst preoperative MGFA class	< 0.001	0.87 (0.81,0.93)	0.141	0.94 (0.87,1.02)
Primary onset-thymectomy time	0.006	0.99 (0.99,0.99)	0.419	0.99 (0.99,1.01)
age	0.275	1.01 (0.99,1.01)	0.372	0.99 (0.99,1.01)
Perioperative MG crisis	0.454	0.80 (0.45,1.43)	0.706	1.13 (0.61,2.09)
Postoperative IM	< 0.001	0.28 (0.21,0.38)	< 0.001	0.29 (0.21,0.41)

### Other outcomes: thymoma vs. nonthymomatous

For the MMS condition, of the initial cohort, 1,046 patients with complete data were included in the multivariate regression analysis after excluding those with missing information. Thymoma did not significantly associate with MMS in the first year after thymectomy (thymoma vs. nonthymomatous, 68.7% vs. 67.2%, adjusted *p* = 0.682), adjusted for age, sex, other immune diseases, primary symptoms, antibody type, preoperative IM, worst preoperative MGFA class, primary onset-thymectomy time, perioperative MG crisis and postoperative IM (Fig. 3C).

A total of 251 patients with complete information from our cohort were selected for the analysis of conversion outcome, with 175 in the thymomatous group and 76 in the nonthymomatous group, were used to explore the correlation between thymic pathology and the conversion rate 2 years after symptom onset. Simple and multivariate logistic regression models revealed that thymomas increased the risk of conversion by 2.37-fold (thymoma vs. nonthymomatous, 45.1% vs. 17.1%, adjusted OR = 2.37 [1.15,4.86], adjusted *p* = 0.019) after adjusting for age, sex, other immune diseases, antibody type, IM history and thymectomy before conversion (Table 4, Fig. 3D).

**Table 4:** Associations between thymomas (except thymic carcinoma) and other outcomes

	**P**	**OR (95%CI)**	**Adjusted p**	**Adjusted OR (95%CI)**
**MMS in the 1 year after thymectomy (***N = 1046)*				
thymoma	0.620	1.07 (0.82,1.39)	0.682	1.07 (0.78,1.47)
Gender	0.728	1.05 (0.81,1.36)	0.146	1.22 (0.93,1.62)
Other immune disease	0.308	0.78 (0.48,1.26)	0.286	0.76 (0.46,1.26)
Primary symptom	0.141	1.22 (0.94,1.58)	0.112	0.79 (0.59,1.06)
Antibody type	0.283	1.14 (0.90,1.45)	0.416	1.11 (0.87,1.41)
Preoperative IM	0.223	0.85 (0.65,1.11)	0.253	0.83 (0.61,1.14)
Worst preoperative MGFA class	0.145	0.95 (0.89,1.02)	0.493	0.97 (0.90,1.05)
Primary onset-thymectomy time	< 0.001	0.99 (0.99,0.99)	< 0.001	0.99 (0.99,0.99)
age	0.158	1.01 (0.99,1.02)	0.729	1.00 (0.99,1.01)
Perioperative MG crisis	0.200	0.67 (0.36,1.24)	0.084	0.57 (0.30,1.08)
Postoperative IM	0.013	1.40 (1.07,1.83)	< 0.001	1.98 (1.43,2.75)
**Transfer from OMG to GMG (***N = 251)*				
Thymoma	< 0.001	3.99 (2.05,7.77)	0.019	2.37 (1.15,4.86)
age	0.456	1.01 (0.99,1.02)	0.199	Ref
Gender	0.748	0.93 (0.60,1.45)	0.194	Ref
Other immune disease	0.074	0.37 (0.13,1.10)	0.794	Ref
IM during 2 years	< 0.001	0.33 (0.18,0.59)	0.031	0.50 (0.26,0.94)
Antibody type	0.008	Ref	0.069	Ref
thymectomy before conversion	0.998	Ref	0.998	Ref

### Thymoma subgroup

**Fig. 4:**
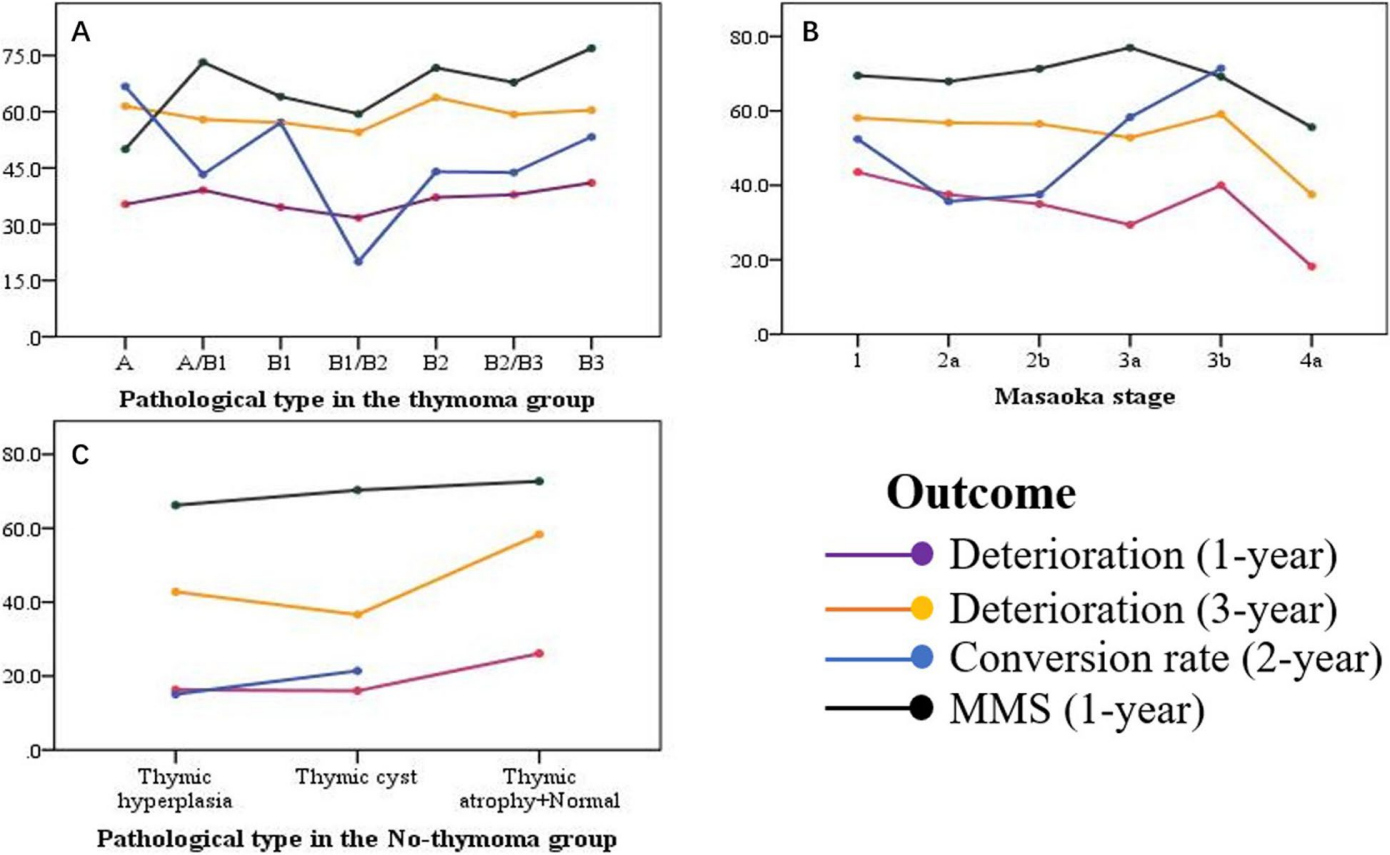
Distribution of deterioration at 1 year or 3 years post-thymectomy, MMS at the first post-thymectomy year and the conversion rate from the OMG to the GMG 2 years after symptom onset according to pathological type in the Thymomatous group (*N* = 623) (**A**), Masaoka stage (*N* = 343) (**B**) and pathological type in the Nonthymomatous group (*N* = 559) (**C**)

The distributions of WHO pathological type and Masaoka stage are summarized in Table 5. With respect to the WHO pathological type of thymoma, no obvious association was observed with deterioration at 1 year (adjusted *p* = 0.069) or 3 years (adjusted *p* = 0.220) after thymectomy or with higher MMS rate in the first post-thymectomy year (adjusted *p* = 0.067). Otherwise, the conversion rate from OMG to GMG within 2 years after symptom onset was not influenced by the pathological type of thymoma (adjusted *p* = 0.606) (Table 6, Fig. 4A).

**Table 5:** Pathology distribution (except thymic carcinoma) in the two subgroups

Thymomatous (*N* = 678)				Nonthymomatous (*N* = 576)	
Pathological type		**Masaoka stage**		**Pathological type**	
A	2.7% (17/623)	**1**	18.1% (62/343)	**Normal**	0.2% (1/559)
A/B1	14.1% (88/623)	**2a**	21.0% (72/343)		
B1	17.5% (109/623)	**2b**	29.2% (100/343)	**thymic hyperplasia**	78.2% (437/559)
B1/B2	6.9% (43/623)	**3a**	19.8% (68/343)		
B2	33.7% (210/623)	**3b**	8.7% (30/343)	**thymic cyst**	3.9% (22/559)
B2/B3	12.2% (76/623)	**4a**	3.2% (11/343)		
B3	12.9% (80/623)	**4b**	0	**thymic atrophy**	17.7% (99/559)
Uncertain	*N* = 55	**Uncertain**	*N* = 335	**Uncertain**	*N* = 17

**Table 6:** Associations between specific pathologies/Masaoka stage and outcomes

	**P**	**OR (95%CI)**	**Adjusted p**	**Adjusted OR (95%CI)**
**Thymoma Pathological type**				
1-year deterioration	0.600	1.03 (0.93,1.13)	0.069	1.17 (0.99,1.40)
3-year deterioration	0.572	1.03 (0.92,1.16)	0.220	1.13 (0.93,1.37)
1-year MMS	0.222	1.08 (0.96,1.21)	0.067	1.18 (0.99,1.41)
Conversion rate	0.793	0.98 (0.81,1.18)	0.606	0.91 (0.64,1.30)
**Thymoma Masaoka stage**				
1-year deterioration	0.130	0.88 (0.74,1.04)	0.944	1.01 (0.82,1.24)
3-year deterioration	0.497	0.94 (0.77,1.13)	0.909	0.99 (0.78,1.24)
1-year MMS	0.844	1.02 (0.85,1.23)	0.579	0.94 (0.76,1.17)
Conversion rate	0.652	1.08 (0.77,1.51)	0.163	1.38 (0.88,2.18)
**Nonthymomatous pathological type**				
1-year deterioration	0.574	Ref	0.806	Ref
3-year deterioration	0.362	Ref	0.654	Ref
1-year MMS	0.736	Ref	0.940	Ref
Conversion rate	0.852	Ref	0.755	Ref

Additionally, for the Masaoka stage, the multivariate regression model revealed no significant differences in deterioration at 1 year (adjusted *p* = 0.944) or 3 years (adjusted *p* = 0.909) after thymectomy, MMS in the first post-thymectomy year (adjusted *p* = 0.579) or the conversion rate (adjusted *p* = 0.163) (Table 6, Fig. 4B). These results were adjusted for age, sex, other immune diseases, primary symptoms, antibody type, preoperative IM, worst preoperative MGFA class, primary onset-thymectomy time, perioperative MG crisis and postoperative IM, post-thymectomy radiotherapy and post-thymectomy chemotherapy.

​However, it is important to note that this analysis was likely underpowered due to the limited subgroup size (WHO pathology, *N* = 623; Masaoka stage, *N* = 343), and the lack of statistical significance should not be interpreted as definitive evidence of the absence of an effect.

### Nonthymomatous subgroup

Most patients in the nonthymomatous subgroup were pathologically diagnosed with thymic hyperplasia (81.1%) (Table 5). Deterioration at 1 year and 3 years post-thymectomy (adjusted *p* = 0.806; 3 years, adjusted *p* = 0.654), MMS at 1 year after thymectomy (adjusted *p* = 0.940) and the conversion rate (adjusted *p* = 0.755) were not influenced by the nonthymomatous WHO pathological type according to the multivariate regression model (Table 6, Fig. 4C).

## Discussion

Our large-sample, multicenter study provides robust support for previous reports indicating that thymomas significantly increase the risk of post-thymectomy deterioration and generalization in patients with MG [5–8]. However, regarding short-term post-thymectomy MMS rate, our findings revealed no significant difference between the thymomatous and nonthymomatous groups. We speculate that this may be attributed primarily to the use of immunoregulatory therapy following thymectomy [25]. Neurologists typically adjust immunosuppressive medication on the basis of individual patients’ postoperative symptoms to ensure minimal manifestation status, especially for those with relatively severe symptoms at discharge. Therefore, the attainment of MMS shortly after thymectomy may not fully reflect the influence of thymoma on MMS outcomes, as adequate immunotherapy may offset the impact of initial disease severity. We attempted to perform a subgroup analysis based on the use of post-thymectomy immunoregulatory medication (PTIM subgroup vs. No PTIM subgroup). However, thymoma still showed no significant association with MMS in either subgroup (PTIM subgroup: adjusted *p* = 0.554; No PTIM subgroup: adjusted *p* = 0.927). The high rate of MMS achievement at one year after thymectomy (70.7% in the PTIM subgroup and 63.3% in the No PTIM subgroup) may have reduced the statistical power to detect a significant effect in this subgroup analysis. Thus, our study has important clinical implications: when neurologists tailor immunoregulatory treatment to achieve MMS, treatment should be guided more by the patient's clinical symptoms rather than concern for the presence of thymoma. Nevertheless, owing to a greater likelihood of deterioration after thymectomy, patients with thymomas should adhere more strictly to medical advice, avoid known exacerbating factors, and maintain the regular use of immunoregulatory medications. Additionally, in patients with OMG and newly diagnosed thymoma, early thymectomy is recommended to reduce the risk of progression to GMG.

With respect to WHO pathological classification and Masaoka staging, nearly 70% of the thymomatous cases fell between WHO types B1 and B2, and the majority were Masaoka stage ≤ 2b. These distributions align with previous studies [26, 27]. Earlier research has suggested that lower WHO classifications and Masaoka stages may be associated with better outcomes [15, 17–20]. Our results do not support this claim. No significant differences were observed in the rates of deterioration, MMS rate, or generalization across different pathological subtypes or clinical stages. There are several potential explanations for this discrepancy. First, pathological classification and staging are determined at the time of thymectomy; however, thymoma characteristics may evolve over time [28]. In particular, in patients with longer intervals between disease onset and thymectomy, the pathology observed at surgery may not accurately reflect the role of the tumor in disease progression. Second, even if pathological differences exist, their clinical significance may diminish following complete tumor resection. In our cohort, the median interval between MG onset and thymectomy was only two months, which may have minimized the potential impact of thymoma characteristics on prognosis. Thus, the potential impact may be limited, as early thymectomy is strongly recommended for all thymoma patients when they are diagnosed with thymoma. On the basis of our findings, we suggest that clinicians managing MG patients with thymomas may not need to focus heavily on the WHO type or Masaoka stage when planning MG treatment. On the other hand, our study was limited to assessing prognosis within three years after thymectomy, which may not capture potential long-term outcomes. However, given the observed association between thymoma and prognosis—where the at 3 years after thymectomy” effect diminished over time (e.g., OR decreasing from 1.59 at one year to 1.40 at three years)—we speculate that even if long-term effects of thymic pathology exist, they are likely to continue diminishing over time.

In the nonthymomatous group, differences in outcomes were also negligible. Thymic hyperplasia was present in the majority of patients (78.2%), with a normal thymus or thymic cysts observed in only 4.1%. Considering patients’ age at the time of thymectomy, many instances of thymic atrophy may have been misclassified as having a normal histology. However, the outcome distribution across pathological subtypes remained consistent, even when thymic atrophy was categorized within the normal group. Therefore, thymic pathology in nonthymomatous MGs also appears to have little prognostic value.

Our study accounted for post-thymectomy radiotherapy and chemotherapy as confounding factors in the thymoma subgroup. Interestingly, we found that both radiotherapy and chemotherapy significantly reduced the risk of clinical deterioration within one year after thymectomy (radiotherapy: OR = 0.28 [0.16, 0.50], p < 0.001; chemotherapy: OR = 0.41 [0.21, 0.80], *p* = 0.009). At three years, chemotherapy remained associated with reduced risk (OR = 0.47 [0.23, 0.95], *p* = 0.035), although the effect of radiotherapy was no longer statistically significant (*p* = 0.387). This finding is ​consistent with​ previous research [29]. Both radiotherapy and chemotherapy are considered effective adjuvant treatments for thymoma [30, 31]. Previous studies have reported that incomplete resection may lead to post-thymectomy myasthenia gravis (MG) development [32]. Radiotherapy and chemotherapy may reduce postoperative deterioration, possibly by addressing residual thymic tissue.

Several limitations must be acknowledged. First, in the conversion analysis, most thymectomies were performed within two years of symptom onset, implying that MG symptoms often develop prior to detectable tumor formation. Therefore, the relationship between thymoma and OMG-to-GMG conversion before thymectomy requires further investigation. Second, the retrospective nature of our study may introduce potential data inaccuracies. For instance, information obtained via telephone follow-up could be subject to recall bias due to reliance on patients’ self-reported and potentially imperfect recollection of their medical history. Moreover, variation in follow-up methods may have introduced measurement bias, particularly as patients—especially those experiencing anxiety—often found it challenging to accurately characterize their disease remission status. This difficulty in precise self-reporting could potentially lead to an overestimation of the association between thymoma and the outcomes examined. Finally, heterogeneity in patient management and data collection protocols across the participating clinical centers may have introduced a source of bias. Future prospective studies and mechanistic research are needed to clarify the complex interactions between thymic pathology and MG prognosis.

## Conclusions

Compared with nonthymomatous cases, thymoma was associated with higher risks of deterioration after thymectomy and a greater rate of conversion from OMG to GMG within 2 years of symptom onset. No significant association was observed between MMS in the 1 year after thymectomy. Furthermore, neither WHO pathological subtype nor Masaoka stage showed a significant correlation with deterioration or MMS outcomes.

## Data Availability

The datasets generated and analyzed during the current study are not publicly available because patients’ private information was included in our dataset but are available from the corresponding author upon reasonable request.

## Abbreviations

MG: Myasthenia gravis
MMS: Minimal manifestation status
OMG: Ocular myasthenia gravis
GMG: Generalized myasthenia gravis
WHO: World health organization
IM: Immunoregulatory medication
MGFA: Myasthenia Gravis Foundation of America clinical classification
HR: Hazard ratio
OR: Odds ratio
PTIM: Post-thymectomy immunoregulatory medication
